# Subchondral and Osteochondral Unit Bone Damage in the Fetlock Region of Sport Horses Using Low-Field MRI: Case Series

**DOI:** 10.3390/ani15233468

**Published:** 2025-12-02

**Authors:** Donatella De Zani, Vanessa Rabbogliatti, Silvia Rabba, Luigi Auletta, Maurizio Longo, Davide D. Zani

**Affiliations:** 1 Department of Veterinary Medicine and Animal Sciences, University of Milan, 26900 Lodi, Italy; vanessa.rabbogliatti@unimi.it (V.R.); luigi.auletta@unimi.it (L.A.); dr.mauriziolongo@gmail.com (M.L.); 2 Freelance, 28075 Novara, Italy; silviarabba77@gmail.com

**Keywords:** equine, metacarpo/metatarsophalangeal joint, bone pathology, bone marrow lesion

## Abstract

Damage to the osteochondral unit is a common cause of lameness in horses. Published descriptions of MRI findings of osteochondral unit and subchondral damage are currently lacking, and only a few reports describe outcomes in sport horses. The aims of this case series retrospective study were to describe different MRI patterns of osteochondral/subchondral pathology in the fetlock joint using low-field MRI and to provide outcome information. A total of 35 sport horses were included. Data regarding detailed clinical history, treatment and outcome were evaluated. MRI identified a total of 39 bone lesions: 14 were limited to the subchondral plate, and 25 involved the whole osteochondral unit. In 12 horses, a fissure was observed. Areas of high signal intensity on STIR sequences and low signal intensity on T1-weighted sequences within the trabecular bone were observed in association with subchondral and osteochondral unit damage. At follow-up, a persistent lameness was observed in 11 horses, all of which presented with a lesion involving the whole osteochondral unit. Different MRI patterns consistent with lesions involving either the entire osteochondral unit or only the subchondral bone plate could be identified using low-field MRI. MRI examination should be recommended in horses with inconclusive radiographic findings. Our results suggest that lesions limited to the subchondral bone are associated with a better prognosis.

## Simple Summary

Osteochondral unit damage is a common cause of lameness in sport horses; however, published descriptions of magnetic resonance findings are limited. This retrospective case series study aimed to characterize magnetic resonance patterns of osteochondral and subchondral pathology in the fetlock joint using a low-field magnetic resonance imaging system and to assess outcomes. Thirty-five sport horses were evaluated, with detailed clinical histories, treatments, and outcomes analyzed. Magnetic resonance identified 39 bone lesions: 14 were confined to the subchondral bone plate, while 25 involved the entire osteochondral unit. Fissures were detected in 12 horses. Lesions were characterized by areas of high signal intensity on fat-suppressed sequences and low signal intensity in T1-weighted images. All 11 horses that remained lame at follow-up had lesions involving the whole osteochondral unit. The study demonstrated that low-field magnetic resonance can differentiate between subchondral plate lesions and more extensive osteochondral damage, especially in cases of inconsistent radiographic findings. Magnetic resonance examination should be suggested in horses with inconclusive radiographic findings.

## 1. Introduction

Injuries to the fetlock joint surfaces are a well-recognized cause of lameness in racehorses [1,2,3,4,5,6] as well as in sport horses [7,8,9,10]. Osseous lesions can result from supraphysiological loads or repetitive strains that exceed the adaptive remodeling capacity of bone [8,9]. In fact, as a response to external stress, an imbalance between bone reabsorption and bone regeneration can occur [9,11,12]. Repetitive bone strain leads to maladaptive remodeling, resulting in trabecular microdamage. Trabecular microdamage can progress to the subchondral bone and other components of the osteochondral unit [3]. The subchondral bone is the osseous region located directly beneath the articular cartilage. It includes the subchondral bone plate—a thin lamellar layer of cortical bone situated below the calcified cartilage and separated from it by the cement line—and the underlying subarticular spongiosa [13]. The osteochondral unit is made by the articular cartilage and the subchondral bone, anchored to each other by the calcified cartilage [14]. Persistent stress on damaged subchondral bone or an osteochondral unit injury can lead to a stress fracture that can ultimately evolve into a complete fracture [9,12].

Conventional radiography is often unable to detect early stages of bone damage, and, in most cases, incomplete acute or subacute fractures can be missed, particularly when relatively late bone responses are absent [7]. It has been shown that magnetic resonance imaging (MRI), including both low- and high-field systems, is a useful tool for diagnosing subtle osseous lesions, such as incomplete fractures and subchondral bone trauma [7,8,10,15,16,17,18]. Moreover, MRI is deemed superior to radiography for diagnosing subchondral pathologies [1,4,6,10,15,19,20]. In a recent study [21], cone-beam (CB) and fan-beam (FB) computed tomography (CT) were superior to MRI in detecting proximal phalanx sagittal groove fissures, but they performed similarly to MRI in detecting third metacarpal/metatarsal parasagittal fractures. Both CBCT and FBCT were unable to detect bone marrow lesions (BMLs) at the fracture site.

On the other hand, some studies evaluated MRI’s ability to detect cartilage lesions and showed that this ability is affected by magnetic field strength, the spatial resolution provided by the coils, and the use of optimized pulse sequences [22]. In one study, Rovel and colleagues [23] reported that cartilaginous lesions of grade 2 or higher can be detected with low-field MRI, whereas Murray and colleagues [24] demonstrated that MRI can be used to assess cartilage and subchondral bone thickness.

Despite the increasing number of articles recognizing the role of MRI in the diagnosis of osteochondral unit damage, few reports still describe the outcome of sport horses with a diagnosis of osteochondral unit pathology of the fetlock region [8,10]. In a recent study, Faulkner et al. [25] described different MRI patterns or combinations of patterns in horses with sagittal groove disease of the proximal phalanx and also provided a detailed description of the progression of MRI patterns during follow-up.

This study aims to describe the case details and MRI findings, including different osteochondral pathology patterns, using low-field MRI, and to report the outcome in sport horses with osteochondral and subchondral injuries of the fetlock joint.

We hypothesized that injuries involving the entire osteochondral unit would have a worse prognosis than injuries limited to the subchondral plate. Therefore, recognition of their localization could enable more accurate diagnosis and prognosis in sport horses.

## 2. Materials and Methods

### 2.1. Inclusion Criteria and Study Design

Clinical records of sport horses referred to the Veterinary Teaching Hospital of the Department of Veterinary Medicine and Animal Sciences, University of Milan (Italy) for MRI examination of the fetlock region between January 2012 and March 2022 were retrospectively reviewed. In this study, horses with a complete clinical history and MRI findings suggesting primary injuries involving the subchondral or osteochondral unit of the third metacarpal/tarsal bone, or the proximal phalanx, were included. Exclusion criteria for the study were the presence of primary concomitant orthopedic lesions, horses with an incomplete clinical record, or horses for which it was not possible to obtain data concerning the follow-up.

### 2.2. Data Recorded

Clinical data recorded and reviewed included athletic use, level of competition, lameness degree and duration, lameness onset, response to diagnostic analgesia performed by the referring veterinarians, radiographic findings, treatments, and outcome. When available, radiographs were retrieved and evaluated by the first author with more than 10 years of experience (D. De Z.). The MRI studies were blindly re-evaluated using a DICOM viewer (Osirix-MD, Pixmeo, Bernex, Switzerland) by two clinicians with more than 10 years of experience in MRI (D. De Z. and D.D.Z). For each horse, a clinical follow-up consisting of a veterinary reevaluation, an owner interview and interrogation of official sports databases (https://www.fise.it/servizi.html, accessed on 28 November 2025, https://www.irha.it/it/, accessed on 28 November 2025, https://ippica.snai.it/risultati, accessed on 28 November 2025) was obtained.

All information concerning the therapeutic plan was recorded.

Lameness was graded by the referring veterinarian using the American Association of Equine Practitioners (AAEP) scale [26]. Prior to MRI examinations, lameness was reassessed by two of the authors (D. De Z. and D.D.Z.) to verify agreement between what was observed and what was reported in the medical history.

Radiographic examination, when performed, consisted of at least four projections, including lateromedial, dorsopalmar/plantar, dorsolateral-palmar/plantaromedial oblique and dorsomedial-palmar/plantarolateral oblique views.

The MRI acquisitions were performed with a low-field system (Vet-MR, Esaote, Genoa, Italy) with horses under general anesthesia, using a dual-phase array coil. The T1- and T2-weighted (T1w and T2w, respectively) and short tau inversion recovery (STIR) sequences were acquired in at least two orthogonal planes. Lesions were classified as subchondral or involving the whole osteochondral unit, with or without a fissure, based on MRI appearance, as described in Table 1.

The anatomical localization of the lesions included: the recognition of the affected bones (PP or MCIII/MTIII); the identification of the location of the lesion in the sagittal plane in terms of dorsal, central, or palmar/plantar (D-C-P) aspect, and in the dorsal plane in terms of the lateral, sagittal, or medial (L-S-M) aspect, as described in Table 1.

The outcome was defined as positive in all subjects that were sound at the time of returning to work and had no recurrence of lameness. For subjects in which lameness persisted or recurred upon resuming training, the outcome was defined as negative. The outcome was assessed for up to 45 weeks.

### 2.3. Statistical Analysis

Clinical and MRI data were recorded in an electronic spreadsheet (Excel^©^ Microsoft for Mac, v. 16.43, Redmond, WA, USA) and then imported into commercial software for statistical analysis (JMP Pro, v. 17.2, SAS Institute, Cary, NC, USA). All analyses were performed by one author (L.A.) Descriptive statistics are reported as mean ± SD or median (range), according to data distribution, or number of subjects (% of the total) within the two outcome categories.

To explore possible associations between clinical variables and the outcome, age, time to referral, time to follow-up, and lameness grade were compared between the positive and negative outcome groups. Differences in age between the two outcome categories were evaluated using a pooled Student’s *t* test after confirming homoscedasticity with Levene’s test. Differences in time to referral and time to follow-up between the two outcome categories were evaluated with the Mann-Whitney’s *U* test. Differences in lameness grade between the two outcome categories were evaluated with the median test. The association between the onset (sudden vs. progressive), the clinical status at the time of presentation (acute vs. chronic), the affected limb, the therapy applied (rest vs. bisphosphonates) and the outcome was assessed with contingency tables, and with either a χ^2^ or Fisher’s exact tests, as appropriate. Significance was set at *p* < 0.05.

To evaluate the ability of MRI features and their interactions (as subsequently specified) to predict a positive outcome, a univariate nominal logistic regression was applied, with significance set at *p* < 0.10. Whenever a variable effect was significant, it was included in a multivariable logistic regression model, along with clinical variables that were significantly different between the two outcomes in the aforementioned analysis, to identify which could be considered an independent predictor of the outcome.

## 3. Results

### 3.1. Horses

A total of 87 MRIs of the fetlock were performed between January 2012 and March 2022. Thirty-five horses met the inclusion criteria. Thirty-nine horses were excluded due to the absence of osseous lesions, 6 for the presence of tenodesmic lesions in association with bony alterations and 7 because it was not possible to obtain follow-up information.

Thirty-five horses were included; 32 horses were used for show jumping, 2 for competitions of doma vaquera and 1 for reining. Except for 1 Quarter Horse used for reining, 1 Andalusian and 1 Pura Raza Espanola used for the doma vaquera, all horses were warmblood breeds, and the median age was 10.5 (6–16) years.

### 3.2. Clinical History

All horses had unilateral lameness; 30 (86%) patients had sudden-onset lameness, whereas 5 (14%) had a history of progressive, worsening lameness. Lameness was localized to the forelimb in 29 cases (83%) and to the hindlimb in six horses (17%). Seven (20%) horses were referred in the acute phase (duration < 3 weeks), whereas 28 (80%) horses were referred later after the beginning of the lameness (range between 4 and 52 weeks of duration). The overall median lameness grade was 2/5 (1–4); the horses that returned to work had a median lameness grade of 2/5 (1–4), whereas the horses that were deemed lame had a median lameness grade of 2.5/5 (1–4), without any difference between the two groups (*p* = 0.28).

Lameness improved significantly following palmar/plantar digital nerve blocks performed just proximally to the ungular cartilages of the foot in 9 horses, following a low 4-point nerve block in 8 horses and following abaxial sesamoid nerve block (palmar/plantar digital nerve blocks performed at the base of the sesamoid bones) in 5 horses. Eleven horses were sound after intra-articular anesthesia of the metacarpo/metatarso-phalangeal joint, while in one horse, an improvement of 80% was observed after intra-articular metatarso-phalangeal joint block. In 1 horse, no blocks were performed because of a suspected fracture.

### 3.3. Radiographic Findings

**Figure 1:**
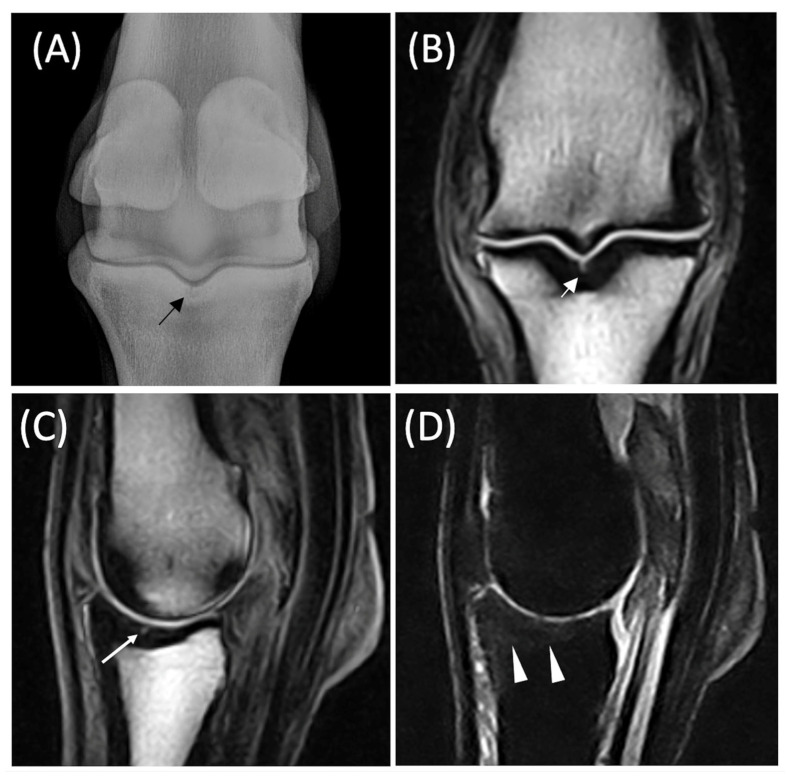
Diagnostic imaging of the right hind fetlock of a 13-year-old gelding used in high-level show jumping competition with a 2/5 lameness of acute onset and a diagnosis of a fissure. In (**A**), Dorsal 20° Proximal-Palmarodistal view of the right hind fetlock (lateral is on the left); a radiolucent ill-defined line (black arrow) is present on the sagittal ridge of the proximal phalanx. In (**B**), Dorsal Turbo 3DT1 image of the same fetlock; a hyperintense line (white arrow) is present in the same location, with involvement of the osteochondral unit. In (**C**), Sagittal Turbo 3DT1 image of the same fetlock. A hyperintense line (white arrow) is present. In (**D**), Sagittal STIR image; the subchondral bone and the spongiosa of the dorsal and central aspect of the sagittal ridge of the proximal phalanx are characterized by a mild increase in signal intensity (white arrowed). The hyperintense line is not visible due to differences in slice thickness between the sagittal sequence.

Radiographs of the fetlock were available for 10 (29%) horses. In 19 horses (54%), radiographic examination of the fetlock was not performed or was performed by the referring veterinarian and was not available for review. In 5 (14%) horses, a complete radiographic examination of the foot was performed due to the positive response to palmar/plantar digital nerve blocks performed just proximally to the cartilage or to abaxial sesamoid block. Out of the 10 horses subjected to radiographic examination of the fetlock, 3 showed no significant alterations. In 2 other horses, changes suggestive of degenerative joint disease of the fetlock were observed. In 2 horses, a subtle, radiolucent, ill-defined line within the subchondral bone of the PP sagittal groove, indicative of a short, incomplete fracture, was observed in the radiographic views (Figure 1A). In 1 horse, a radiolucent lesion was observed affecting the medial articular surface of the PP. In another horse, the radiolucent area was associated with the medial condyle of the MCIII, accompanied by dorsal periosteal reactions. In 2 other horses, periosteal proliferations were noted in the proximal-dorsal aspect of the proximal phalanx.

In 3 cases, the only appreciable finding in the dorso-palmar view was subchondral sclerosis of the proximal aspect of the PP.

### 3.4. MRI Findings

All 35 horses had at least one area of high signal intensity on all sequences within the subchondral plate or the osteochondral unit. Nineteen subjects had the lesion localized on the PP (15 in a forelimb and 4 in a hindlimb), 15 on the MCIII, 1 on both PP and MCIII, 1 on both PP and MTIII, 1 on the MTIII, and a total of 39 lesions were detected. Twelve lesions (31%) were localized at the sagittal groove of the PP, mainly in the central aspect (8 lesions); 2 lesions were localized at the centro-dorsal aspect of the sagittal groove, 1 at the dorsal aspect and 1 at the centro-plantar aspect. Seven lesions (18%) were detected at the central aspect of the medial articular surface of the PP, 15 (38%) at the medial condyle of the MCIII. Five lesions (13%) were classified as Palmar Osteochondral Disease (POD) [17].

**Figure 2:**
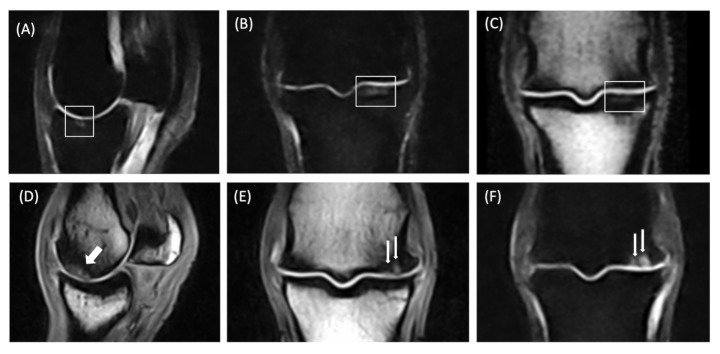
Magnetic resonance findings of the left front fetlock of an 11-year-old gelding used for high-level show jumping competition, diagnosed with an osteochondral lesion involving the subchondral bone of the medial articular surface of the proximal phalanx (**A**–**C**) (white frame). In (**A**), Sagittal plane short tau inversion recovery. In (**B**), Dorsal plane short tau inversion recovery. In (**C**), Dorsal plane Turbo 3DT1 image. A linear transverse hyperintense area is present in the subchondral bone plate in all sequences. The perilesional trabecular bone is characterized by a very mild high signal on STIR images and a heterogeneous low signal on Turbo 3DT1 images. Magnetic resonance images of a 10-year-old Selle Français horse used in medium-level show jumping competition diagnosed with a lesion involving the osteochondral unit of the dorso-central aspect of the medial condyle. The horse was referred for 2/5 lameness of the left front fetlock, which was abolished by intra-articular block of the metacarpophalangeal joint (**D**–**F**). In (**D**), Sagittal plane Turbo 3DT1. In (**E**), Dorsal plane Turbo 3DT1. In (**F**), Dorsal plane STIR. Lateral is on the left. Two high signal areas are present in the centro-dorsal aspect of the subchondral bone of the medial condyle of MCIII on STIR and Turbo 3DT1 sequences (white arrows). In all images, the hyperintense areas involved the subchondral bone plate and the subchondral trabeculae. The trabecular bone of the medial condyle is heterogeneously hypointense on T1 sequences. A cartilage defect is seen in association with the hyperintense lesions described.

The increased signal was limited to the subchondral plate (Figure 2A–C) in 13 (33%) lesions, whereas in 26 (67%) lesions the area of high signal intensity involved the osteochondral unit (Figure 2D–F). Moreover, in 16 out of 26 lesions involving the osteochondral unit, an associated cartilage defect was detected. A fissure, characterized by a linear high signal intensity in all sequences (Figure 1B), was evident within the high signal intensity in 12 horses (34%), and 11 of these extended into the trabecular bone (Table 2).

The fracture line was located at the level of the sagittal groove of the PP in 7 horses, whereas in the remaining 5 horses, the linear high signal intensity was located at the dorsal aspect of the medial condyle of the MCIII.

Areas of BML, characterized by high signal intensity on STIR sequences and low signal intensity on T1 sequences within the trabecular bone, were observed in association with subchondral and osteochondral unit damage (Figure 2D–F) in 26 of 35 horses (74%). Small bone marrow lesions were localized in the trabecular bone surrounding the subchondral and osteochondral unit abnormalities in 17 horses; in 4 horses, BML extended from the dorsal to the palmar/plantar aspect of the sagittal groove, whereas in 2 horses it extended from the dorsal to the palmar/plantar aspect of the medial articular surface of the proximal phalanx. Bone marrow lesions less than 1 cm were detected in the medial articular surface of the proximal phalanx in 3 horses with osteochondral unit damage of the medial condyle of the third metacarpal/metatarsal bone.

In 9 cases (27%), no signal alterations of the trabecular bone were observed.

In 13 horses, trabecular bone sclerosis was observed surrounding subchondral (4 horses) or osteochondral (9 horses) lesions. Bone sclerosis was characterized by low signal intensity of the trabecular bone on T1w sequences.

### 3.5. Treatment

All horses received 8 weeks of box rest and controlled work of variable duration ranging from 4 to 12 weeks (mean 10 weeks).

Five horses were treated with systemic administration of bisphosphonates; all had a diagnosis of osteochondral unit involvement, and 3 of them had a fissure. One horse with lesions within the subchondral plate was treated with an intra-articular injection of hyaluronic acid. Details about the treatment can be found in Supplementary Table S1.

The median time to the final follow-up was 32 weeks (range: 12–40 weeks).

### 3.6. Outcome

Of the 35 horses included in the study, 23 (66%) were sound at the time of the clinical reevaluation: 21 returned to their previous sport level, and 2 competed at a lower level. Twelve horses (34%) were still lame at the time of the follow-up; 5 of these horses were retired from competition due to the lameness’s persistence (Table 2).

Overall, the median follow-up time was 30 weeks (range 12–45); sound horses that returned to work displayed a significantly (*p* = 0.0005) shorter (26 weeks; 12–45 weeks) follow-up time compared to still lame horses (40 weeks; 20–45).

The mean age of the positive outcome group was 10 ± 2 years, and the mean age of the negative outcome group was 13 ± 2 years, with sound horses significantly younger than still lame horses (*p* < 0.0001). The time to referral was 8 weeks (range 2–52) for horses that returned to work and 6 weeks (range 2–52) for still-lame horses. The group of horses that were sound at the clinical reevaluation had a median lameness grade of 2/5 (range 1–4), whereas the still lame group had a median lameness grade of 2.5/5 (range 1–4), without any difference between the two groups (*p* = 0.28). No association between the affected limb and the outcome could be detected (*p* = 0.56). The onset (*p* = 0.77), time to referral (*p* = 0.84), clinical status at presentation (*p* = 0.83), and medical treatment applied (*p* = 0.77) did not differ between the two outcomes.

At the univariate analysis, neither the bone affected (*p* = 0.57), nor the L-S-M (*p* = 0.99) or D-C-P (*p* = 0.83) localizations alone were associated with the outcome. Similarly, neither the interaction of the bone affected with the L-S-M (*p* = 0.76), the D-C-P localization (*p* = 0.38), nor both (*p* = 0.47) effects were associated with the outcome. Two out of 6 horses with an area of BML extending from the dorsal aspect to the palmar/plantar aspect of the articular surface of the PP or of the MCIII/MTIII condyle were lame at the clinical reevaluation; the other four horses returned to compete at the same level. The presence of a fissure line was not associated with the outcome (*p* = 0.51).

The subchondral plate (*p* = 0.002) and osteochondral unit lesions (*p* = 0.007) appeared associated with outcome. All horses with subchondral lesions returned to work with a positive outcome, whereas all horses in the negative outcome group at clinical reevaluation had osteochondral involvement, and 2 of them had a fissure. Overt cartilage loss was also observed in 7/24 (29%) sound horses.

The multivariable nominal logistic regression included, as explanatory variables, the presence of subchondral plate lesions, the presence of osteochondral unit lesions and age of the horse. The model was highly significant, explaining 65% of the variance, with a non-significant lack-of-fit statistic (*p* = 0.91). Only the age of horses retained the ability to predict the outcome (*p* = 0.002), whereas neither the presence of a subchondral unit nor osteochondral lesion was an independent predictor of the outcome (*p* = 0.99 for both).

## 4. Discussion

Subchondral and osteochondral lesions are well-known and recognized pathologies in human medicine. Even if subchondral lesions have a better prognosis than lesions involving the whole osteochondral unit, they should not be underestimated, as they can lead to subchondral cysts, overlying cartilage collapse, and osteonecrosis [27]. Osteochondral lesions, on the other hand, can lead to the development of chronic conditions, including arthritis and joint degeneration [28].

Differentiation between subchondral and osteochondral unit lesions can be made using MRI, based on recognition of specific patterns, which helps formulate an accurate prognosis and choose a suitable therapy [29,30].

In our study, distinct patterns of subchondral or osteochondral pathology were identified using low-field MRI. While low-field MRI demonstrated lower sensitivity for detecting cartilage lesions than high-field MRI or arthro-CT, it provides a reliable assessment of trabecular bone lesions and subchondral abnormalities, especially when using a variety of sequences [22,23,24,31,32,33,34].

In our study, the differentiation between lesions involving the subchondral bone plate and the whole osteochondral unit was achieved using a combination of STIR and T1w sequences in different planes. Short tau inversion recovery sequences allowed the recognition of BMLs, whereas a combination of T1w, STIR, and T2w sequences was mandatory for the detection of subchondral and cartilage injuries [22,23,24,31,32,33,34]. Cartilage defects were observed only when associated with trabecular bone damage; indeed, in the present study, no lesions of the cartilage alone were detected. A possible explanation for this result is that the sensitivity of low-field MRI for detecting partial cartilage defects is poor [31,32,34]. Even if trabecular bone abnormalities can highlight the area of cartilage injury, the high signal associated with bone pathology can negatively affect the detection of cartilage loss due to partial-volume averaging [32]. In recent years, it has been shown that chondral lesions are often underestimated, even with high-field MRI [33].

Except for the horse with a suspected fracture, diagnostic anesthetic blocks were performed to identify the source of pain in the fetlock region in 20 of the 35 horses included. In the remaining 14 horses, diagnostic blocks localized the lameness to the foot or pastern region. In these horses, there was likely a proximal diffusion of local anesthetic solution due to the proximal injection site (2 cm proximal to the ungular cartilages) and the amount of the local anesthetic used (>2 mL per site) [35].

Similar to Dyson, the present study reported an overall soundness of 66% [7]. However, another study reported a worse prognosis with a lower percentage of soundness (32%), attributed to the development of osteoarthritis or to underestimated cartilage lesion severity on low-field MRI [8].

In horses included in the current work, no signs of osteoarthritis were observed. However, it should be noted that the injuries observed by Dyson [7] and Gold [8] were localized only at the level of the sagittal groove of the PP.

Smith et al. [36] reported a positive outcome in 92% of horses with a short incomplete parasagittal fracture of PP that underwent surgical repair, suggesting that surgical repair should be considered as the optimal choice in these cases. In a study by Lipreri et al. [10], 50% of horses with documented osseous trauma returned to their previous athletic levels, regardless of surgical or conservative treatment, but no differentiation was made between lesion types. In our study, the presence of a fissure did not affect return to work or soundness. A subtle radiolucent line suggestive of an incomplete fracture was observed in only two of 15 horses that underwent a complete radiographic examination, while subchondral sclerosis of the proximal aspect of the PP was the only change detected in another case, confirming that short incomplete fractures can be missed using plain radiography, especially in acute cases [37,38]. As suggested by other authors, if subtle changes in subchondral bone density are observed, further investigation should be considered to prevent catastrophic injuries and the progression to a complete fracture [7,16]. Although bone scintigraphy can detect early bone remodeling and increased bone activity caused by exercise-induced stress, it has poor specificity [7]. In fact, despite increased radiopharmaceutical uptake (IRU) associated with proximodorsal short fractures of PP, which was pathognomonic and well recognized [16], Dyson et al. [7] reported that IRU was more diffuse. In human medicine, even though bone scan has been considered the reference standard for tibial stress injuries for many years, some limitations are now recognized, such as the presence of abnormal IRUs in asymptomatic patients or the presence of clinical signs in people with negative bone scans [39]. Magnetic resonance investigation, as well as bone scintigraphy, permits an early detection of bone injuries. In addition, MRI allows evaluation of both bone and soft tissue and should be considered the gold standard for horses with suspected short, incomplete fractures. In human medicine, MRI is considered the gold standard diagnostic technique for the classification and/or grading of both stress and insufficiency fractures [39,40,41]. Moreover, based on MRI findings, a prompt and specific treatment can be selected to improve patient prognosis [41,42]. Faulkner et al. [25] identified some MRI patterns of progression of sagittal groove disease that should be considered a potential prodromal sign of worsening of the pre-existing pathology in non-racing sports horses.

No correlation was observed between lesion localization or identification of subchondral versus osteochondral lesions, but all horses that returned to work had lesions involving the subchondral plate, while horses that were still lame had lesions involving the whole osteochondral unit. The lack of a statistically significant correlation could be due to the limited number of cases included in the study.

Neither the lameness onset and duration nor the lameness grade at time of presentation was associated with the outcome.

In our study, age was associated with the outcome: younger horses were more likely to be sound at the time of the clinical reevaluation. In humans, marrow stem cell depletion in bone has been demonstrated, along with an accumulation of reactive oxygen species related to aging, which can negatively interfere with bone modeling and remodeling [43]. Alterations in bone modeling can lead to a deterioration of subchondral bone and a decrease in trabecular thickness [43,44]. In contrast to these results, some studies in both humans [45] and horses [46] did not find any age-related changes in subchondral bone morphology. Although the theory of a possible relationship between bone remodeling and aging can explain our results, further studies are necessary to verify the association between age and outcome.

The present study inherits some limitations. The osteoarticular lesions displayed different localization in a relatively small sample. Indeed, a possible influence of the localization over the latero-medial and dorso-palmar planes on the outcome might be hypothesized. Unfortunately, the two categorization systems used to summarize lesion localization might have led to data dispersion, thereby reducing the statistical power to detect such an influence. Similarly, the lack of effect of the bisphosphonate should be considered with care, since it was applied only in five horses. Different treatments may have influenced the outcome [36], such as surgical repair or calcium dobesilate administration [47,48]. It was not possible to perform a gross or histological examination of the involved joints; however, given the current literature, we consider it likely that the chondral lesions observed in our cases could be effective, even if a possible underestimation of severity should be considered. Indeed, it would be interesting to obtain an anatomopathological validation of the MRI features described in this study.

## 5. Conclusions

In conclusion, in the present study, a differentiation between lesions involving the whole osteochondral unit and lesions isolated to the subchondral bone plate was achieved using low-field MRI. Radiographic investigation was unable to detect injuries of the subchondral plate, of the osteochondral unit, or to highlight the presence of an overt fracture line in most cases. Based on the results obtained in the present study and what is reported in the literature [7,10,15,16], MRI examination should be recommended when subchondral damage of the fetlock joint is suspected despite the absence of findings on radiographs. A low percentage of horses displaying lesions to the osteochondral unit returned to soundness, whereas all horses in which only the subchondral bone was involved returned to work.

## Figures and Tables

**Table 1:** MRI features and localization system. The table describes the main MRI appearances of the detected lesions, the locations for image analysis and the localization of the lesions observed.

	MRI Consistent Lesion Features	Additional Possible MRI Findings
Subchondral lesion	Arcuated or linear or irregular localized hyperintensity within the subchondral bone in all sequences; the subchondral black line, due to chemical shift artifacts, is normal in shape, signal and thickness; no indentation/depression of the overlying cartilage	Perilesional or diffuse BML
Osteochondral Unit lesion	Arcuated or irregular localized hyperintensity in all sequences extended from the cartilage layer to the subchondral plate, with disruption of the black line; depression of the cartilage into bone can be observed, as well as a cartilage defect	Perilesional or diffuse BML; fissure
Fissure	Linear hyperintensity with proximodistal orientation extending from the cartilage layer to the subchondral or the trabecular bone; the black line is interrupted; depression of the cartilage into bone can be observed, as well as a cartilage defect	Perilesional bone densification and/or diffuse BML
BML	Ill-defined low signal intensity of the trabecular bone on T1w images and ill-defined areas of high signal intensity on T2w or STIR images	Subchondral or osteochondral lesions; fissure; bone densification
Bone sclerosis	Ill-defined low-signal intensity areas in all sequences directly in contact with the subchondral bone	BML; fissure; subchondral or osteochondral lesions
**Localization on the transverse plane**		
Dorsal	Between the dorsal cortex of PP or MCIII/MTIII and the dorsal border of the collateral ligament fossae	
Palmar/Plantar	Between the palmar/plantar border of the collateral ligament fossae and the palmar/plantar cortex of PP or MCIII/MTIII	
Central	Between the dorsal and the palmar/plantar borders of the collateral ligament fossae	
**Localization on the dorsal plane**		
Sagittal	Involvement of the sagittal ridge of MCIII/MTIII or of the sagittal groove of PP	
Medial	Involvement of the medial condyle of MCIII/MTIII or of the medial aspect of PP	
Lateral	Involvement of the lateral condyle of MCIII/MTIII or of the lateral aspect of PP	

MRI: magnetic resonance imaging; BML: bone marrow lesion; MCIII: third metacarpal bone; MTIII: third metatarsal bone; PP: proximal phalanx; T1w: T1-weighted; T2w: T2-weighted; STIR: short tau inversion recovery.

**Table 2:** For each case, the affected limb, the characteristics of the lameness (onset, duration, grade), the outcome, and the MRI findings, i.e., the localization of the hyperintense lesions, appreciable in both T1-weighted and STIR sequences, and the involvement of the subchondral plate or the osteochondral unit. The presence of a fissure is indicated.

Case	Fetlock	Onset	Lameness Duration	Lameness Grade	Localization	MRI Lesions			
						Fissure	Subchondral Lesion	Osteochondral Unit Lesion	Outcome
1	LF	progressive	2 weeks	4	medial articular surface PP	no	no	yes	negative
2	LH	acute	4 weeks	3	medial articular surface PP	no	yes	yes	sound at work, same level
					medial condyle, dorsal	no	yes	no	
3	RH	acute	4 weeks	3	sagittal groove PP, centro-plantar	yes	no	yes	negative
4	RH	acute	8 weeks	2	sagittal groove PP, central	no	yes	no	sound at work, same level
5	LF	acute	52 weeks	2	medial articular surface PP	no	yes	no	sound at work, same level
6	LH	progressive	8 weeks	2	POD lateral	no	yes	no	sound at work, same level
					POD medial	no	yes	no	
7	LF	acute	2 weeks	3	medial condyle, dorsal	no	no	yes	negative
8	LH	acute	52 weeks	1	sagittal groove PP, central	yes	no	yes	negative
9	LF	acute	26 weeks	3	medial condyle, dorsal	yes	no	yes	negative
10	RF	acute	8 weeks	2	sagittal groove PP, centro-dorsal	no	no	yes	sound at work, same level
11	RF	acute	2 weeks	4	sagittal groove PP, dorsal	yes	no	yes	sound at work, same level
12	LF	acute	16 weeks	2	medial articular surface PP	no	no	yes	negative
13	RF	acute	8 weeks	4	sagittal groove PP, central	no	no	yes	negative
14	RH	acute	16 weeks	2	sagittal groove PP, central	yes	no	yes	sound at work, same level
15	RF	acute	2 weeks	2	medial condyle, dorsal	yes	no	yes	negative, retired from work
16	LF	acute	8 weeks	3	medial condyle, dorsal	no	no	yes	sound at work, same level
17	RF	acute	4 weeks	3	sagittal groove PP, central	yes	no	yes	sound at work, low level
18	RF	acute	4 weeks	2	medial condyle, dorsal	yes	no	yes	sound at work, same level
19	LF	acute	8 weeks	2	medial condyle, dorsal	no	yes	no	sound at work, same level
20	RF	acute	2 weeks	2	medial articular surface PP	no	yes	no	sound at work, same level
21	RF	acute	8 weeks	2	medial condyle, dorsal	no	no	yes	negative, retired from work
22	RF	acute	8 weeks	3	sagittal groove PP, central	no	yes	no	sound at work, same level
23	RF	acute	8 weeks	2	sagittal groove PP, centro-dorsal	no	yes	no	sound at work, same level
24	RF	acute	8 weeks	1	sagittal groove PP, central	yes	no	yes	sound at work, same level
25	LF	progressive	2 weeks	2	medial articular surface PP	yes	yes	yes	sound at work, same level
					medial condyle, dorsal	yes	no	yes	
26	LF	progressive	4 weeks	2	sagittal groove PP, central	yes	no	yes	negative, retired from work
27	LF	acute	2 weeks	2	medial condyle, dorsal	yes	no	yes	sound at work, same level
28	RF	progressive	12 weeks	2	medial condyle, centro-palmar	no	yes	no	sound at work, same level
29	LF	acute	8 weeks	1	POD lateral	no	no	yes	negative, retired from work
					POD medial	no	no	yes	
30	LF	acute	4 weeks	2	medial condyle, dorsal	no	no	yes	sound at work, same level
31	RF	acute	4 weeks	2	POD lateral	no	yes	no	sound at work, same level
32	LF	acute	4 weeks	2	medial articular surface PP	no	yes	no	sound at work, same level
33	RF	acute	2 weeks	4	medial condyle, dorsal	no	no	yes	sound at work, same level
34	LF	acute	4 weeks	3	medial condyle, dorsal	no	no	yes	negative, retired from work
35	LF	acute	8 weeks	2	medial condyle, dorsal	no	no	yes	sound at work, low level

PP: proximal phalanx; RF: right front; LF: left front; RH: right hind; LH: left hind; POD: palmar osteochondral disease.

## Data Availability

Data are available from the corresponding author upon reasonable request due to restrictions (privacy or ethical reasons).

## Supplementary Materials

The following supporting information can be downloaded at: https://www.mdpi.com/article/10.3390/ani15233468/s1, Table S1. For each case are summarized the lame limb, the characteristics of the lameness (onset, duration, grade), the outcome and the MRI findings, referring to the localization of the hyperintense lesions appreciable in both T1 weighted and STIR sequences and the involvement of the subchondral plate or the osteochondral unit. The presence of a fissure is indicated. PP: proximal phalanx; RF: right front; LF: left front; RH: right hind; LH: left hind. Treatment (rest, medical treatment and period of controlled exercise) is reported, as well as outcome.

## Abbreviations

The following abbreviations are used in this manuscript:

CB	cone-beam
CT	computed tomography
D-C-P	dorsal, central, palmar/plantar
BML	Bone Marrow Lesion
FB	fan-beam
IRU	increased radiopharmaceutical uptake
L-S-M	lateral, sagittal, medial
MCIII	Third Metacarpal Bone
MRI	Magnetic Resonance Imaging
MTIII	Third Metatarsal Bone
POD	palmar osteochondral disease
PP	Proximal Phalanx
STIR	short tau inversion recovery
T1w	T1-weighted
T2w	T2-weighted
