# Supplementary material for: Subchondral and Osteochondral Unit Bone Damage in the Fetlock Region of Sport Horses Using Low-Field MRI: Case Series
Source: Animals (Basel). 2025 Dec 2;15(23):3468. doi: 10.3390/ani15233468 (PMC12691481; doi:10.3390/ani15233468)
Supplement: Supplementary file 1 [file animals-15-03468-s001.zip › animals-3956319-supplementary.pdf]

						MRI lesions			Treatment			Outcome
Case	Fetlock	Onset	Lameness duration	Lameness grade	Localization	Fissure	Subchondral lesion	Osteochondral unit lesion	Rest (weeks)	Additional therapy	Controlled work (weeks)	
1	LF	progressive	2 weeks	4	medial articular surface PP	no	no	yes	8		12	lame
2	LH	acute	4 weeks	3	medial articular surface PP	no	yes	yes	8		10	sound at work, same level
					medial condyle, dorsal	no	yes	no	8		4	sound at work, same level
3	RH	acute	4 weeks	3	sagittal groove PP, centro-plantar	yes	no	yes	8		10	lame
4	RH	acute	8 weeks	2	sagittal groove PP, central	no	yes	no	8		8	sound at work, same level
5	LF	acute	52 weeks	2	medial articular surface PP	no	yes	no	8		10	sound at work, same level
6	LH	progressive	8 weeks	2	POD lat	no	yes	no	8		6	sound at work, same level
					POD med	no	yes	no	8		6	
7	LF	acute	2 weeks	3	medial condyle, dorsal	no	no	yes	8		12	lame
8	LH	acute	52 weeks	1	sagittal groove PP, central	yes	no	yes	8		12	lame
9	LF	acute	26 weeks	3	medial condyle, dorsal	yes	no	yes	8		10	lame
10	RF	acute	8 weeks	2	sagittal groove PP, centro-dorsal	no	no	yes	8		10	sound at work, same level

11	RF	acute	2 weeks	4	sagittal groove PP, dorsal	yes	no	yes	8		8	sound at work, same level
12	LF	acute	16 weeks	2	medial articular surface PP	no	no	yes	8		12	lame
13	RF	acute	8 weeks	4	sagittal groove PP, central	no	no	yes	8		12	lame
14	RH	acute	16 weeks	2	sagittal groove PP, central	yes	no	yes	8		10	sound at work, same level
15	RF	acute	2 weeks	2	medial condyle, dorsal	yes	no	yes	8	systemic administration of bisphosphonates	12	lame, ritired from work
16	LF	acute	8 weeks	3	medial condyle, dorsal	no	no	yes	8	systemic administration of bisphosphonates	8	sound at work, same level
17	RF	acute	4 weeks	3	sagittal groove PP, central	yes	no	yes	8	systemic administration of bisphosphonates	10	sound at work, low level
18	RF	acute	4 weeks	2	medial condyle, dorsal	yes	no	yes	8	systemic administration of bisphosphonates	10	sound at work, same level
19	LF	acute	8 weeks	2	medial condyle, dorsal	no	yes	no	8	Intra-articular injection of Hyaluronic Acid	8	sound at work, same level
20	RF	acute	2 weeks	2	medial articular surface PP	no	yes	no	8		8	sound at work, same level
21	RF	acute	8 weeks	2	medial condyle, dorsal	no	no	yes	8	systemic administration of bisphosphonates	12	lame, ritired from work
22	RF	acute	8 weeks	3	sagittal groove PP, central	no	yes	no	8		12	sound at work, same level

23	RF	acute	8 weeks	2	sagittal groove PP, centro-dorsal	no	yes	no	8	10	sound at work, same level
24	RF	acute	8 weeks	1	sagittal groove PP, central	yes	no	yes	8	8	sound at work, same level
25	LF	progressive	2 weeks	2	medial articular surface PP	yes	yes	yes	8	10	sound at work, same level
					medial condyle, dorsal	yes	no	yes	8	10	
26	LF	progressive	4 weeks	2	sagittal groove PP, central	yes	no	yes	8	12	lame, ritired from work
27	LF	acute	2 weeks	2	medial condyle, dorsal	yes	no	yes	8	10	sound at work, same level
28	RF	progressive	12 weeks	2	medial condyle , centro-palmar	no	yes	no	8	8	sound at work, same level
29	LF	acute	8 weeks	1	POD lat	no	no	yes	8	12	lame, ritired from work
					POD med	no	no	yes	8	12	
30	LF	acute	4 weeks	2	medial condyle, dorsal	no	no	yes	8	10	sound at work, same level
31	RF	acute	4 weeks	2	POD lat	no	yes	no	8	8	sound at work, same level
32	LF	acute	4 weeks	2	medial articular surface PP	no	yes	no	8	10	sound at work, same level
33	RF	acute	2 weeks	4	medial condyle, dorsal	no	no	yes	8	10	sound at work, same level
34	LF	acute	4 weeks	3	medial condyle, dorsal	no	no	yes	8	12	lame, ritired from work

35	LF	acute	8 weeks	2	medial condyle, dorsal	no	no	yes	8	12	sound at work, low level

Table 1: For each case are summarized the lame limb, the characteristics of the lameness (onset, duration, grade), the outcome and the MRI findings, referring to the localization of the hyperintense lesions appreciable in both T1 weighted and STIR sequences and the involvement of the subchondral plate or the osteochondral unit. The presence of a fissure is indicated. PP: proximal phalanx; RF: right front; LF: left front; RH: right hind; LH: left hind. Treatment (rest, medical treatment and period of controlled exercise) is reported, as well as outcome.
